# Shotgun Metagenomic Characterization of Acne Microbiota Before and After Treatment with a Topical Biotechnological Phytocomplex: Understanding Skin Dysbiosis

**DOI:** 10.3390/microorganisms14040915

**Published:** 2026-04-18

**Authors:** Adrià Cruells, Cristina Eguren, Aymée Robainas Barcia, Helena Martínez, Mohammed Sharaf, Carlos Ruiz, Antonio Sánchez-Baos, Nerea Carrón, Lola Bou, Montse Pérez, Raúl De Lucas, Aurora Guerra-Tapia

**Affiliations:** 1 Microomics Systems S.L., 08980 Sant Feliu de Llobregat, Spain; adria.cruells@microomics.com (A.C.); antonio.sanchez@microomics.com (A.S.-B.); nerea.carron@microomics.com (N.C.); 2 Clínica Eguren S.L.P., 28009 Madrid, Spain; c.eguren@outlook.com; 3 Laboratorio Reig Jofre, 08970 Sant Joan Despí, Spain; helena.mar.ser@gmail.com (H.M.); msharaf@reigjofre.com (M.S.); cruiz@reigjofre.com (C.R.); 4 Consulta de Dermatología, 08006 Barcelona, Spain; dralolabou@gmail.com; 5 Clínica Dermatológica de Moragas, 08034 Barcelona, Spain; peluttrufa@gmail.com; 6 Hospital Universitario La Paz, 28046 Madrid, Spain; rauldelucas@gmail.com; 7 Consulta Dermatóloga Guerra, 28027 Madrid, Spain; aurora@auroraguerra.com

**Keywords:** acne vulgaris, microbiome, microbiota, shotgun, *Cutibacterium acnes*, *Malassezia*, quorum sensing

## Abstract

This study assessed the impact of a topical phytocomplex on the acne skin microbiota, encompassing bacterial, fungal, and phage communities. Skin samples obtained from participants exhibiting a positive response to the treatment were analyzed using high-throughput sequencing and bioinformatic approaches including taxonomic profiling, metagenome assembly, functional annotation, and phage identification. Results showed that after treatment, microbial diversity increased, reflecting a more balanced microbial composition. *Cutibacterium acnes* levels were reduced, particularly virulent IA1/IA2 phylotypes, whereas non-pathogenic or unclassified strains increased. Opportunistic pathogens such as *Klebsiella pneumoniae* were no longer detected, and beneficial genera including *Psychrobacter* and *Dermabacter* were enriched. Functional analysis showed reduced virulence- and biofilm-related pathways, alongside enhanced tryptophan metabolism, SCFA production, lipid synthesis, and riboflavin and folate biosynthesis. Fungal populations, dominated by *Malassezia*, became more evenly distributed, with notable post-treatment reductions in *M. arunalokei*, *Exophiala spinifera*, and *Wickerhamomyces anomalus*. Phage populations mirrored bacterial changes, with enrichment of Cutibacterium-associated phages post-treatment. These findings demonstrate that the phytocomplex promotes functional rebalancing of the skin microbiota by reducing pathogenic features while maintaining ecosystem stability. The inhibition of quorum sensing, potentially mediated by N-acyl-homoserine lactone acetylation, emerged from metabolic pathway annotation as a hypothetic key mechanism impairing bacterial communication and virulence associated with acne vulgaris.

## 1. Introduction

Acne vulgaris, is a chronic inflammatory disorder of the skin that affects the pilosebaceous unit [1]. The condition appears when hair follicles become obstructed by a combination of sebum and death cells [2]. Acne can lead to physical complications such as scarring or post-inflammatory hyperpigmentation and psychological as well as anxiety and/or depression [3]. Adolescents are the most affected population, although adults may also experience the condition [4,5]. Epidemiological studies estimate that approximately 85% of individuals between the ages of 12 and 24 will experience at least one episode of acne, with some continuing to have persistent acne into adulthood [1,3,4].

Current treatment options for acne, such as topical retinoids and benzoyl peroxide, primarily address the symptoms rather than the underlying disease [6]. In severe cases, systemic antimicrobial therapy is approved, despite potential drawbacks including antimicrobial resistance and other adverse effects [7]. Emerging therapeutic strategies are increasingly focused on modulating the skin microbiome [8]. These approaches target the role of microbial communities in acne pathogenesis and aim to restore a balanced microbiome, thereby improving patients’ quality of life [9].

The main components of the skin microbiome are bacteria, fungi, viruses and mites, all of which contribute to skin health and functionality [10,11]. The human skin is second only to the gut in terms of bacterial density, with an approximate density of 104 to 106 bacteria per square centimeter and over 200 genera characterized [12].

More than 40 bacterial genera have been described on the skin, with the phyla Actinobacteria, Firmicutes, Proteobacteria, and Bacteroidetes being the most prevalent [13]. Among these phyla, Gram-positive bacteria are predominant, with *Cutibacterium* (formerly *Propionibacterium*), *Staphylococcus*, and *Corynebacterium* representing the most commonly detected genera together with other typical skin taxa [8,14].

*Cutibacterium acnes* plays a central role in acne pathogenesis. It is a Gram-positive bacterium commonly found in the human microbiome, including the skin and gut [14] which acts as an opportunistic pathogen in various skin disorders and is the most prevalent bacterial species associated with acne vulgaris [15]. *C. acnes* has been recently subdivided into six main phylogenetic groups or phylotypes: IA1, IA2, IB, IC, II and III [16].

Phylotype I includes the most pathogenic strains (IA1 and IA2). Especially the IA1 phylotype, display strong virulent properties, including biofilm formation, production of proteolytic enzymes, lipases, porphyrins, and stimulation of Th17/Th1-type immune responses [17].

The skin microbiota plays a crucial role in maintaining skin homeostasis, some microorganisms are associated with a healthy skin, maintaining the acidic pH of the epidermis by releasing free fatty acids [10]. However, under certain circumstances, such as acne, this homeostasis is lost. Sebaceous-rich areas of the skin exhibit increased microbial activity, including enhanced sebum utilization via lipases and the formation of biofilms [14]. Moreover, *C. acnes* contributes with hyperseborrhea, as it is implicated in lipogenesis and sebum production, stimulating the sebaceous glands and sebum synthesis via the corticotropin-releasing hormone (CRH)/CRH receptor pathway. In particular, CRH augments the synthesis of sebaceous lipids and induces IL-6 and IL-8 release by sebocytes, mediated by the CRH receptor [18]. The microbiota can also contribute to skin inflammation through activation of pro-inflammatory pathways, while simultaneously reducing populations of bacteria with protective roles, such as *Corynebacterium granulosum*, which may negatively affect *C. acnes* biofilm formation [8]. Also, quorum sensing inhibition has been described as an interesting potential mechanism for microbiome modulation, as it regulates virulence factors in bacteria associated with acne vulgaris [19].

Fungi are increasingly recognized as key modulators of acne and related skin disorders [10]. *Malassezia* spp. are known to be involved in the pathogeneses of various dermatological conditions, including seborrheic dermatitis (SD). These species dominate sebaceous skin niches and metabolize lipids efficiently, producing free fatty acids and pro-inflammatory metabolites that can exacerbate follicular occlusion and local immune activation [20,21]. Its lipase activity contributes to the excessive breakdown of sebum triglycerides, while cell wall components stimulate keratinocyte pattern-recognition receptors, amplifying cytokine release and sustaining inflammation [22].

*Malassezia* spp. have also been correlated with acne [23]. The hypothesis of a potential protective role when occupying enough of the skin niche to prevent colonization by other microbial species is under discussion. The most recently described species, *M. arunalokei*, has been found to have a high relative abundance on the forehead and cheek compared to the scalp, a niche which is currently occupied by other *Malassezia* species [24].

Previous studies from our group have demonstrated that acne treatments can reduce disease severity while modulating the skin bacterial population [25].

In this study we hypothesize whether changes in the composition and diversity of skin bacterial and fungal communities are associated with clinical improvements in mild-to-moderate acne following 8 weeks of treatment with a facial gel-cream containing an antibiofilm biotechnological phytocomplex, by comparing pre- and post-treatment microbiome profiles through a shotgun metagenomic analyses. By addressing this question, we seek to explore associations between changes in the skin microbiota and clinical improvement in acne within a responder-defined cohort, generating preliminary hypothesis-generating insights of microbiota involvement in acne pathophysiology or identification of therapeutic microbial targets.

## 2. Materials and Methods

### 2.1. Study Design

The analyzed samples correspond to a subgroup of patients from a study previously conducted by our group between October 2022 and February 2023 [25].

The study was an open-label, prospective clinical study, in 44 subjects with mild–moderate acne who were treated for 8 consecutive weeks (56 days). The study included five visits, at baseline (D0), after 7 days (D7), after 14 days (D14), after 28 days (D28), and after 56 days (D56) of use of the product.

The study product, Vincobiosis^®^ acneic (Laboratorio Reig Jofre S.A., Barcelona, Spain), was a facial cream gel containing Canonia allysis^®^ (*C. sinensis* and *M. citrifolia* callus lysate), niacinamide 4.00% and succinic acid 2.00%.

### 2.2. Study Population

The study initially enrolled 44 patients. Following two dropouts, 42 patients remained clinically evaluable. Shotgun metagenomic sequencing was conducted in a subgroup of 20 patients who exhibited a favorable clinical response.

#### 2.2.1. Inclusion Criteria

Subjects were eligible for inclusion if they met all the following criteria:

- Age between 12 and 35 years.
- Caucasian men and women presenting oily skin with mild to moderate acne-prone conditions.
- Acne evolution time of more than 2 months.
- Discontinuation of topical anti-acne or sebum-regulating products at least 7 days prior to the start of the study.
- Ability and willingness to sign the informed consent form.
- No application of any products to the experimental area on the first day of the study.

#### 2.2.2. Exclusion Criteria

Subjects were excluded from participation if any of the following conditions applied:

- Use of antibiotics, antihistamines, corticosteroids, beta-blockers, retinoids, azelaic acid, or any anti-acne therapy that was not discontinued at least 15 days before study initiation.
- Use of oral contraceptives or any other form of hormonal treatment.
- Application of topical products other than those provided by the study on the experimental area, or exposure of the area to sunlight or UVA radiation during the study period.
- History of allergy to cosmetic products, skin hyperreactivity, oncologic disease, recent surgery or treatment in the study area, or autoimmune disorders.

All patients were instructed to apply the product to the face, morning and evening, after regular cleansing with a supplied neutral gel and massage until completely absorbed.

### 2.3. Study Assessments

#### 2.3.1. Samples

Skin microbiota analysis was carried out at Microomics Systems S.L. (Barcelona, Spain).

Samples were collected on the initial day (D0) and after 56 days of product application (D56) to evaluate microbiota changes in the affected area. Microbiome samples were obtained from the skin selected area using Corneofix^®^ adhesive strips. Each strip was applied to the skin for 1 min, removed with sterile forceps, and placed into a cryovial. The procedure was repeated by applying a second strip to the same area and pooling both samples in the same vial. Samples were stored at −20 °C until shipment on dry ice to the Microomics Systems S.L. laboratory, where they were kept at −80 °C until analysis.

#### 2.3.2. Shotgun Analysis

Skin microbiota analyses were conducted on a subset of 40 samples collected at baseline (D0) and after 56 days of treatment (D56) from 20 participants who exhibited a favorable clinical response, as evidenced by the improvements described by De Lucas et al. (2024) [25]. Each patient acted as its control, but a non-responder control group was included.

Libraries were performed by Illumina True seq (Illumina Inc., San Diego, CA, USA) and were sequenced using NovaSeq (Illumina Inc., San Diego, CA, USA). The sequencing reads were sequenced in Illumina true seq, obtaining pair-end sequences. Adapter sequences were removed from the reads using Trimmomatic v0.39 [26]. Reads with a median quality score below 30 [Phred score] were discarded using BBduk (BBTools v38.96) [27]. High-quality reads were then mapped to the human genome [GRCh38] using Bowtie2 v2.5.1 [28]. All reads underwent trimming and read quality filtering, and any read pair with at least one read mapping to the human genome was removed.

Prokaryotic taxonomic assignment was performed using Sylph v0.4.1 [29] against the Genome Taxonomy Database (GTDB) [30]. Reads were assembled with MEGAHIT v1.2.9 [31] and annotated using Prokka v1.14.6 [32], with functional analyses performed against enzymatic commission (EC) [33] and MetaCyc [34] databases.

*Cutibacterium* phylotyping was performed by first filtering out all non-*Cutibacterium* reads using Bowtie2 v2.5.1 [28], then assembling the remaining reads into *Cutibacterium* assemblies. Taxonomic assignment was conducted using Centrifuge v1.0.4 [35], including only sequences classified at the species level within the genus *Cutibacterium* [all former *Propionibacterium* sequences were renamed as *Cutibacterium*]. Assembly quality was assessed with CheckM v1.2.2 [36], and phylotypes were classified based on sequencing types [STs] following the classification of McDowell et al. Antibiotic resistance genes were analyzed against the Resistance Gene Database [37]. For eukaryotic taxonomic analyses, bacterial reads were first removed using Centrifuge [35] against a bacterial RefSeq database [38]. The remaining non-prokaryotic reads were assembled with MEGAHIT v1.2.9 [31], and taxonomy was assigned using Kaiju v1.9.2 [39] against a RefSeq database [38] of eukaryotic sequences. Phages were annotated using Sylph v0.4.1 [29] against a manually curated viral database.

Raw data are available in ENA with the accession number PRJEB104013.

### 2.4. Statistical Analysis

Diversity analyses were normalized by rarefaction. Alpha diversity was calculated using observed taxa and Pielou’s evenness index. Statistical analyses for alpha diversity were performed using a generalized linear model (GLM), with a negative binomial model for Richness and beta-regression for Evenness. Beta diversity was calculated using Bray–Curtis distances, with statistical significance assessed through PERMANOVA and PERMDISP tests. Differential abundance analyses were conducted using the NBZIMM algorithm. Metabolic pathway and gene function analyses were performed using a negative binomial model. In all statistical models, the formula used was *y* ~ *treatment*, where *y* represents the variable of interest. The *p*-value adjustment was performed using the Benjamini–Hochberg false discovery rate (BH). All analyses were performed in R version 4.3 [35].

## 3. Results

### 3.1. Demographic Analysis

A total of 44 patients were initially included in the study, with a mean age of 20.45 years, and 70.45% women. Two patients dropped out from the study for reasons unrelated to the study, and no patients were excluded. Therefore, 42 patients were included in the clinical evaluation, of whom a responder-defined subgroup of 20 patients was selected for shotgun metagenomic analysis.

The demographic data of the participants tested, including age, sex, and ethnicity, showed no statistically significant differences within or between groups. This indicates that the groups were comparable in these baseline characteristics, and therefore, the effects observed in the study are unlikely to be attributed to confounding variables other than the treatment itself.

### 3.2. Prokaryotic Domain

**Figure 1:**
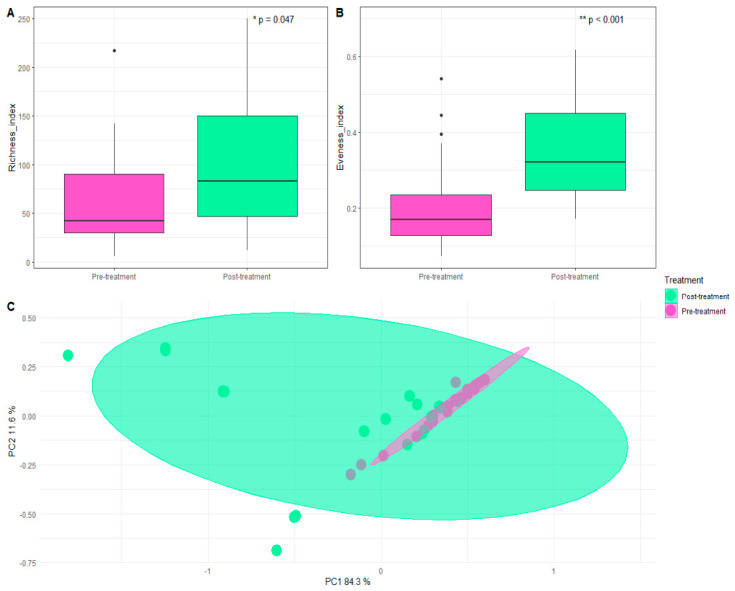
Bacterial diversity: alpha and beta diversity among samples. Alpha diversity was calculated using observed taxa and Pielou’s evenness index. Statistical analyses for alpha diversity were performed using a generalized linear model (GLM), with a negative binomial model for Richness and beta-regression for Evenness: (**A**) The X-axis corresponds to the study pre-treatment (*n* = 20) and post-treatment (*n* = 20) groups, while the Y-axis to sample richness. Boxplots summarize the distribution of values within each group: the central line represents the median, the box corresponds to the interquartile range (IQR), and the whiskers extend to 1.5 times the IQR; points outside this range indicate potential outliers. (**B**) The X-axis represents the study groups, while the Y-axis shows the evenness of each sample. The boxplots summarize the distribution of values within each group: the central line represents the median, the box corresponds to the IQR, and the whiskers extend to 1.5 times the IQR; points outside this range indicate potential outliers. (**C**) Beta diversity was assessed using Bray–Curtis dissimilarity. The X-axis (PC 1) corresponds to the first principal coordinate and the Y-axis (PC 2) to the second. Each point represents a sample, and ellipses denote the 95% confidence interval around the group centroid. * *p* < 0.05; ** *p* < 0.001. Total sample size was *n* = 40; pre-treatment group (*n* = 20) and post-treatment group (*n* = 20).

In the prokaryotic analysis, a total of 754 distinct taxa were identified across all samples. The diversity analyses revealed a significant increase in alpha diversity, including both richness and evenness (Richness, *p* = 0.047; Evenness, *p* < 0.001). Beta diversity revealed significant sample clustering. Additionally, significant clustering by individual participants was detected. PERMDISP analysis indicated a significant dispersion of the data (Figure 1).

**Figure 2:**
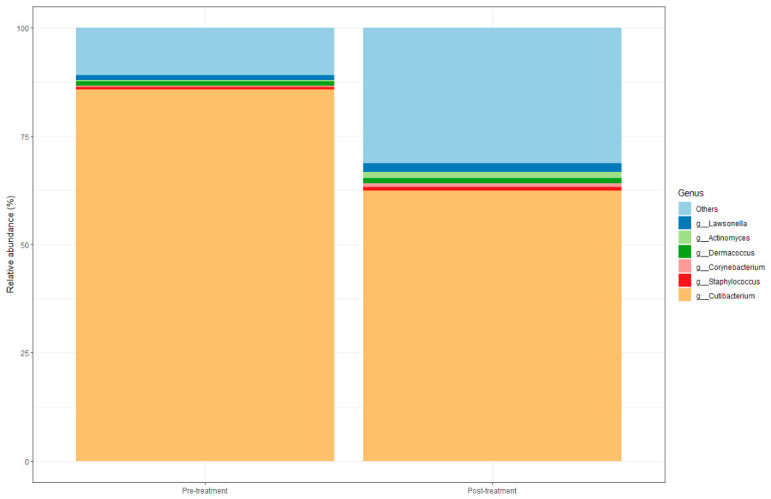
Bacterial composition. The figure depicts the most abundant bacterial genera across samples. The X-axis indicates the study pre-treatment and post-treatment groups, while the Y-axis displays the relative abundance of each genus. Total sample size was *n* = 40; pre-treatment group (*n* = 20) and post-treatment group (*n* = 20).

Within the prokaryotic profiles, the phylum Actinomycetota was the most abundant in the samples before the treatment, followed by Pseudomonadota, Bacillota, and Bacteroidota. Post-treatment, a significant decrease in Actinomycetota was observed, accompanied by relative increases in Pseudomonadota and Bacillota. The *Staphylococcus*/*Cutibacterium* ratio increased (0.009 vs. 0.03). Differential abundance analysis identified 99 taxa with significant changes. Among these, the most decreased species belonged to the genera *Cutibacterium*, *Arachnia*, and *Propionimicrobium* (Figure 2).

**Figure 3:**
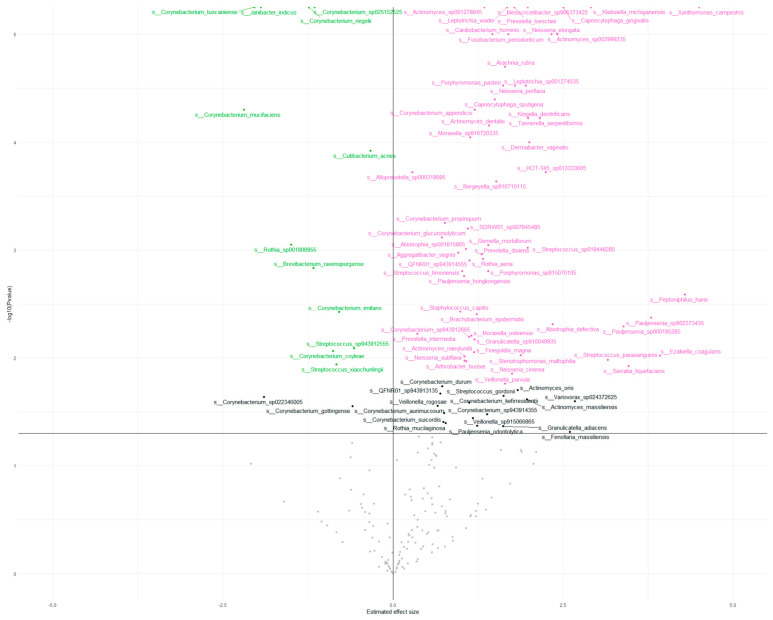
Bacterial differential abundance. Volcano plot represents the differential abundance analysis of bacterial species between the study groups. Each dot corresponds to a bacterial species. The X-axis indicates the log_2_ fold change, and the Y-axis shows the −log_10_ of the adjusted *p*-value. Gray dots represent species with non-significant differences. Black dots indicate species with statistically significant differences [adjusted *p* < 0.05]. Green dots denote species that are significantly more abundant in the pre-treatment group according to the adjusted *p*-value, whereas pink dots indicate species significantly more abundant in the post-treatment group.

From the genus *Cutibacterium*, a significant reduction from *C. acnes* in the post-treatment group has also been detected. In the post-treatment group, significant increases were observed in the genera *Psychrobacter* and *Dermabacter*, as well as in the species *Staphylococcus capitis* and *Brachybacterium epidermidis* (Figure 3).

**Figure 4:**
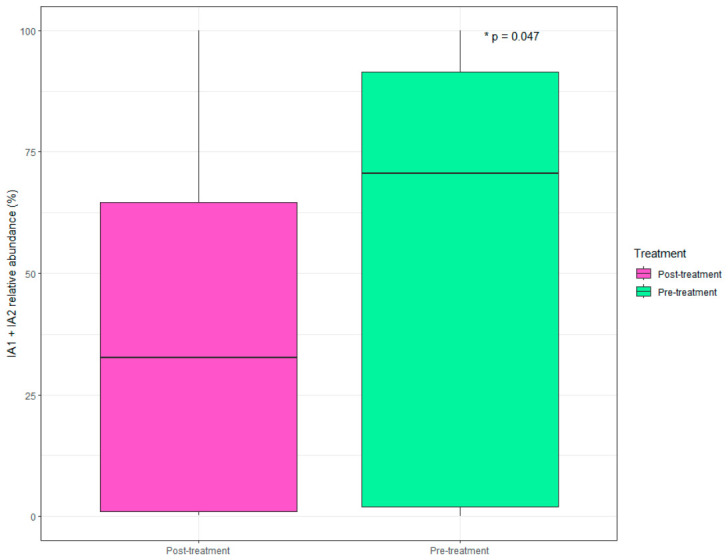
Phatogenic phylotypes abundance. Relative abundance of *C. acnes* phylotypes (IA1, IA2) among samples, by study group. The X-axis represents the study groups, while the Y-axis shows the abundance of each sample (%). The boxplots summarize the distribution of values within each group: the central line represents the median, the box corresponds to the interquartile range (IQR), and the whiskers extend to 1.5 times the IQR; points outside this range indicate potential outliers. * *p* < 0.05.

The *Cutibacterium* genus Metagenome-Assembled Genomes (MAGs) showed a decrease in the abundance of virulent phylotypes IA1 and IA2 post-treatment compared to pre-treatment samples. The sum of these pathogenic lineages was significantly lower (*p* < 0.05) after treatment. In all samples, a co-occurrence of multiple *Cutibacterium* phylotypes was detected, including both virulent and non-virulent types (Figure 4). Additionally, environmental phylotypes were identified in both pre- and post-treatment groups, suggesting their stable presence within the community. No resistance genes were detected in any *Cutibacterium* MAGs. * *p* < 0.05.

Statistical models from prokaryotic analyses are available on Supplemental Materials S1.

A total of 472 significant metabolic pathways were identified in the study, of which 286 remained significant after *p*-value correction. After excluding pathways with not known effect on skin health, post-treatment samples showed increased activity in pathways related to tryptophan metabolism [TRPSYN-PWY], short-chain fatty acid [SCFA] production [CENTFERM-PWY], triacylglycerol synthesis [TRIGLYN-PWY], and riboflavin and folate biosynthesis [RIBOSYN2-PWY/FOLSYN-PWY]. General metabolic maintenance pathways, predominant in pre-treatment, were reduced post-treatment (Table 1).

A total of 746 significant enzymatic functions were detected, of which 559 remained significant after *p*-value correction. In the post-treatment group, increased activity was observed for enzymes such as aldehyde reductases [EC 1.1.1.135/1.1.1.194], NAD[P]H quinone oxidoreductases [EC 1.6.5.10/1.6.5.7], asparaginase [EC 3.5.1.11], and various acetyltransferases [EC 2.3.1.20/2.3.1.79]. In contrast, the pre-treatment group predominantly showed enzymatic functions associated with sterol esterases [EC 3.1.1.41], carboxylesterases [EC 3.1.1.99], muramidases [EC 3.2.1.17], aminopeptidases [EC 3.4.11.18], and metalloproteases [EC 3.4.24.55] (Table 2).

Statistical models from functional analyses are available on Supplemental Materials S2.

### 3.3. Fungal Domains

**Figure 5:**
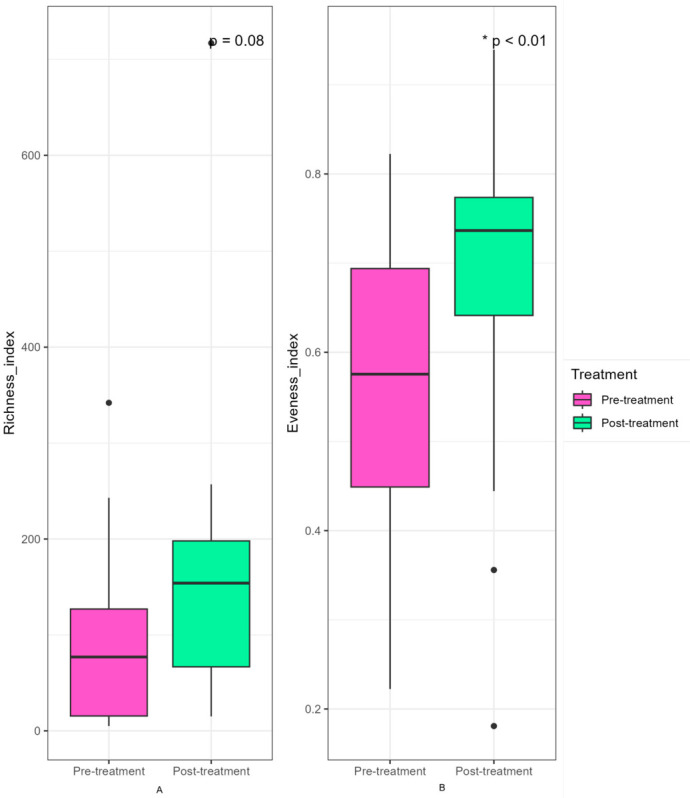
Fungal diversity. This figure illustrates the alpha diversity among the samples faced by study pre-treatment and post-treatment groups. Alpha diversity was calculated using observed taxa and Pielou’s evenness index. Statistical analyses for alpha diversity were performed using a generalized linear model (GLM), with a negative binomial model for Richness and beta-regression for Evenness. (**A**) The X-axis represents the study groups, while the Y-axis shows the richness of each sample. The boxplots summarize the distribution of values within each group: the central line represents the median, the box corresponds to the interquartile range (IQR), and the whiskers extend to 1.5 times the IQR; points outside this range indicate potential outliers. (**B**) The X-axis represents the study groups, while the Y-axis shows the evenness of each sample. The boxplots summarize the distribution of values within each group: the central line represents the median, the box corresponds to the IQR, and the whiskers extend to 1.5 times the IQR; points outside this range indicate potential outliers. * *p* < 0.05. Total sample size was *n* = 40; pre-treatment group (*n* = 20) and post-treatment group (*n* = 20).

In the Fungal domain, a total of 1834 taxa were identified across samples. Post-treatment samples showed an increase in alpha diversity, which was statistically significant for evenness (*p* = 0.01) but not for richness (*p* = 0.08). Beta diversity did not show significant clustering between groups; however, it was revealed that each participant harbored an independent and individualized eukaryotic population (Figure 5).

Statistical models from microbial analyses are available on Supplemental Materials S3.

**Figure 6:**
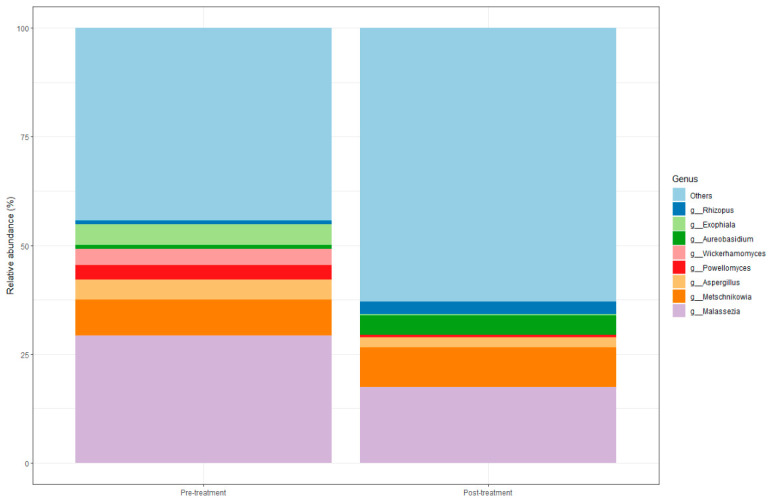
Fungal composition. The figure depicts the most abundant fungal genera across samples. The X-axis indicates the study groups, while the Y-axis displays the relative abundance of each genus.

Within the Fungal domain, the genus *Malassezia* was identified as the most prevalent in both groups. A reduction in *Malassezia* abundance was observed in the post-treatment group, with *M. arunalokei* showing the greatest decrease. Similarly, *Exophiala spinifera* and *Wickerhamomyces anomalus* were also reduced post-treatment. In contrast, the genera *Aureobasidium*, *Zymoseptoria*, and *Debaryomyces* showed increased representation in the post-treatment group (Figure 6).

### 3.4. Phage Domain

Regarding the phagueome, a correlation between relative abundances of *C. acnes* phylotypes and *Cutibacterium* phages [formerly *Propionibacterium* phages] pre- and post-treatment was found, being statistically significant (*p* < 0.05) for certain phages (PHL082M03, P14, Moyashi, PHL041M10, PHL092M00) related with bacteria survival and film formation potential (Table 3). A significant post-treatment increase was detected for PHL082M03 and P14, whereas Moyashi, PHL041M10 and PHL092M00 were significantly reduced.

Statistical models from microbial analyses are available on Supplemental Materials S4.

## 4. Discussion

The effect of the treatment has revealed a shift in the microbiome composition and function. Diversity analysis suggests that the phytocomplex promotes a microbiome equilibrium with a redistribution of microbial taxa rather than the dominance of a few groups. This pattern is consistent with other reports where an external modulation with topical formulations induces a community restructuring that reflects microbial competition and resource reallocation [40,41]. Genetic differentiation analyses support a strong subject-specific microbiota profile, revealing that the skin microbiome of each volunteer was redistributed rather than replaced [42].

A reduction in Actinomycetota and an enrichment of the phylum’s Pseudomonadota and Bacillota were observed. These community-level microbial shifts were also observed in treatments that modified skin hydration or lipid composition [43,44]. The decline in Actinomycetota suggest a reduction in some lipid-dependent genera such as *Cutibacterium* and *Arachnia*. Post-treatment enrichment of *Psychrobacter* and *Dermabacter*, associated with stress resistance and lipid metabolism, also support that the treatment might reestablish commensal bacteria, as these taxa have been described as more abundant in healthy skin [45].

MAG analyses of the *Cutibacterium* genus revealed a significant reduction in the pathogenic phylotypes IA1 and IA2 after treatment, whereas non-pathogenic [phylotype II] or unclassified phylotypes increased. This result suggests that the phytocomplex did not eradicate *Cutibacterium* populations but rather favored less virulent subtypes, a phenomenon previously documented through comparative genomic analyses of *C. acnes* [46]. The absence of resistance genes in *Cutibacterium* MAGs further supports that the microbial changes were caused by ecological rather than selective pressures for resistant strains, thus confirming that the treatment promotes a balanced microbial state without disrupting the overall ecosystem integrity [47].

Functional analysis indicated a relative reduction, after treatment, in predicted metabolic pathways associated with biofilm formation, cell adhesion, porphyrin production, and other virulence-related functions inferred from pathogenic *Cutibacterium acnes* phylotypes. In contrast, post-treatment samples showed enrichment in predicted pathways related to tryptophan metabolism, short-chain fatty acid (SCFA) biosynthesis, triacylglycerol synthesis, and riboflavin and folate cofactor biosynthesis. These pathways have been described in the literature as being involved in redox balance, lipid metabolism, and cofactor availability, and may reflect shifts in microbial metabolic potential relevant to host–microbe interactions [48]. Conversely, pre-treatment samples mainly display pathways related to general metabolic maintenance and hydrolytic enzyme function [muramidases, aminopeptidases, metalloproteases], which are often involved in cell wall remodeling and degradation of complex substrates. Taken together, these observations suggest that, prior to treatment, the skin microbiome may have exhibited a functional profile enriched in turnover- and stress-associated processes, whereas post-treatment samples displayed a relative enrichment of inferred anabolic and cooperative metabolic functions [49]. However, these interpretations are based on metagenomic inference and should be regarded as indicative of potential functional shifts rather than direct evidence of altered metabolic activity. Notably, an increased activity of acetyltransferases after treatment suggests the potential suppression of quorum sensing through N-acyl-homoserine lactone acetylation, a mechanism known to disrupt bacterial communication and virulence regulation [50]. Nevertheless, this interpretation requires direct functional validation. Some values in the model were inflated as a result of the low abundance of the function, primarily in pre-treatment samples where overall diversity is reduced. While an increase in these functions is still observed, the effect size may be artificially amplified.

The fungal domain followed similar but subtler trends. Diversity analysis revealed that fungal populations, dominated by the genus *Malassezia*, became more evenly distributed after treatment. This result confirms the correlation between some *Malassezia* species and acne skin [51].

Particularly interesting was the finding of a post treatment significant reduction in *M. arunalokei*. This species, the most recently described within the *Malassezia* genus, has not previously been associated with seborrheic dermatitis or acne. However, it has predominantly been isolated from sebum rich areas such as the cheeks and forehead [24]. As no prior information was found in the literature, the present study constitutes the first documented association between *M. arunalokei* and acne-prone, sebum-rich skin environments, suggesting a potential ecological role for this species in dysbiotic conditions.

Furthermore, post treatment decreases in *Exophiala spinifera* and *Wickerhamomyces anomalus*, indicated a modulation of the fungal community toward less lipid-dependent or opportunistic taxa [52,53,54]. Simultaneous increases in *Aureobasidium* and *Debaryomyces* suggest replacement by commensal yeasts with different metabolic requirements, consistent with adaptive mycobiome responses observed following alterations in surface lipid content or microbial cross-talk [55,56].

Bacteriophages are essential as part of skin microbiome and have been shown to play a role in human skin health and disease, some increasing bacterial virulence [57]. Marinelli et al. suggested that bacteriophage-mediated killing of *C. acnes* can attenuate inflammatory pathways induced by these bacteria [58]. Our findings might confirm this hypothesis and that of Liu et al. where prey–predator relationship between bacteria and phage may have a role in modulating the composition of the microbiota [59]. Some *C. acnes* phages were detected in both groups, suggesting that phage analysis could be an efficient tool to evaluate different *C. acnes* strains within the microbiome. However, more studies are needed at phage level.

Some limitations should be considered when interpreting the results of this study. Most importantly, the absence of a negative or control arm represents a methodological limitation that, while allowing internal comparisons within the analyzed cohort, might constrain causal interpretation. In addition, some effect size estimates may appear elevated, likely reflecting the low representation of certain inferred functions in one of the study groups; however, this does not invalidate the observed associations, which should be interpreted strictly within an exploratory framework. Although the sample size was relatively limited, it was sufficient to identify reproducible trends within the responder-defined subgroup analyzed.

Taken together, these considerations indicate that the present findings should be regarded as preliminary and hypothesis-generating. Further studies incorporating larger and more heterogeneous cohorts, appropriate control groups, and long-term follow-up will be required to assess the robustness and persistence of the observed microbiome changes. Particularly, longitudinal investigations will be necessary to determine whether these microbial shifts persist following treatment discontinuation, revert toward baseline states, or reflect transient adaptations of the skin microbial ecosystem.

## 5. Conclusions

This study presents an exploratory analysis of the associations between treatment with a biotechnological phytocomplex and changes in the predicted virulence profile of the acne skin microbiota. The treatment significantly reduced the abundance of the most pathogenic *C. acnes* phylotypes (IA1 and IA2), while promoting an increase in the non-pathogenic phylotype II.

A significant modulation in the relative abundance of phages infecting *Cutibacterium* has also been observed, suggesting that microbial shifts in the skin environment may influence the dynamics between bacteriophages and their bacterial hosts.

Additionally, a marked reduction in *Malassezia* spp. was observed, particularly *M. arunalokei*, which is reported here for the first time with significant relative abundance in acne-prone skin. Furthermore, a downregulation of inflammatory and metabolic pathways associated with biofilm formation, cell adhesion, porphyrin production, and virulence factor expression was detected. The observed enrichment of acetylation-related functions supports the hypothesis that N-acyl-homoserine lactone acetylation may represent a putative mechanism for quorum sensing interference, potentially affecting *C. acnes* communication and virulence regulation.

Collectively, the results indicate that treatment with the phytocomplex may be associated with changes consistent with a functional rebalancing of the skin microbiota, including a relative reduction in inferred pathogenic features, while preserving ecosystem stability.

## Figures and Tables

**Table 1:** Differentially abundant metabolic pathways identified between the pre- and post-treatment groups. The table reports the estimated effect size (reflecting the magnitude of the difference in predicted pathway abundance between pre- and post-treatment samples; positive values indicate higher abundance after treatment, whereas negative values indicate reduced abundance), the standard error (SE), and the corresponding *p*-values and Benjamini–Hochberg (BH) adjusted *p*-values. Metabolic pathways with adjusted *p* < 0.05 were considered significantly modulated after treatment.

Pathway	Estimated Effect Size	SE	*p*-Value	*p*-Adjusted
TRPSYN-PWY	0.30	0.09	0.0062	0.0311
CENTFERM-PWY	0.97	0.31	0.0063	0.0316
TRIGLYN-PWY	1.45	0.39	0.0019	0.0149
RIBOSYN2-PWY	1.69	0.34	0.0001	0.0020
FOLSYN-PWY	1.41	0.33	0.0006	0.0059

**Table 2:** Differentially abundant enzymatic functions identified between pre- and post-treatment groups. The table shows the estimated effect size (the magnitude of the difference in the abundance of enzymatic functions between the pre- and post-treatment groups; positive: increase after treatment, negative: reduction after treatment), standard error (SE), and the corresponding *p*-values and *p*-adjusted values (BH correction). Pathways with adjusted *p* < 0.05 were considered significantly modulated after treatment.

EC	Estimated Effect Size	SE	*p*-Value	*p*-Adjusted
1.1.1.135	52.52	0.11	<0.0001	<0.0001
1.1.1.194	51.88	0.12	<0.0001	<0.0001
1.6.5.10	1.14	0.28	0.0009	0.0065
1.6.5.7	1.85	0.31	0.0002	0.0026
3.5.1.11	46.53	0.12	<0.0001	<0.0001
2.3.1.20	52.34	0.09	<0.0001	<0.0001
2.3.1.79	1.92	0.50	0.0014	0.0080
3.1.1.41	−0.27	0.10	0.0157	0.0490
3.1.1.99	−0.65	0.22	0.0092	0.0335
3.2.1.17	−0.96	0.32	0.0086	0.0325
3.4.11.18	−0.16	0.04	0.0030	0.0156
3.4.24.55	−1.46	0.31	0.0002	0.0022

**Table 3:** Differentially relative abundance of significant *C. acnes* phages identified between pre- and post-treatment groups. The table shows the mean abundances in both groups and the corresponding *p*-values and *p*-adjusted values (BH correction).

EC	Pre-Treatment Mean Abundance	Post-Treatment Mean Abundance	*p*-Value	*p*-Adjusted
PHL082M03	0.0000	0.1274	<0.0001	<0.0001
P14	0.0401	0.2184	0.0002	0.0013
Moyashi	0.1400	0.0047	0.0034	0.0121
PHL041M10	0.3125	0.0000	0.0070	0.0226
PHL092M00	0.4607	0.0254	0.0284	0.0782

## Data Availability

The original contributions presented in this study are included in the article/Supplementary Materials. The raw data presented in the study are openly available in ENA with the accession number PRJEB104013. Further inquiries can be directed to the corresponding author.

## Supplementary Materials

The following supporting information can be downloaded at: https://www.mdpi.com/article/10.3390/microorganisms14040915/s1, S1: Differential models for Prokaryotes; S2: Statistical models for functional models; S3: Differential models for Eukaryotes; S4: Differential models for Virus.
